# [Fe^III^Cl(TMPPH_2_)][Fe^III^Cl_4_]_2_: A Stand-Alone Molecular Nanomedicine That Induces High Cytotoxicity by Ferroptosis

**DOI:** 10.3390/molecules29112495

**Published:** 2024-05-24

**Authors:** Xiao Wang, Jia-Hao Feng, Chun-Mei Zeng, Ze-Sheng Zhang, Feng-Lin Cao, Wen-Hua Zhang, Jin-Xiang Chen, David J. Young

**Affiliations:** 1 College of Chemistry, Chemical Engineering, and Materials Science, Soochow University, Suzhou 215123, China; wxxx0225@163.com (X.W.); 20214209084@stu.suda.edu.cn (C.-M.Z.); 20224209184@stu.suda.edu.cn (Z.-S.Z.); xingyue5435@163.com (F.-L.C.); 2 NMPA Key Laboratory for Research and Evaluation of Drug Metabolism, Guangdong Provincial Key Laboratory of New Drug Screening, School of Pharmaceutical Sciences, Southern Medical University, Guangzhou 510515, China; 13078100247@163.com; 3 Glasgow College UESTC, University of Electronic Science and Technology of China, Chengdu 611731, China; david.j.young@glasgow.ac.uk

**Keywords:** single-molecular nanomedicine, porphyrin ligand, chemodynamic therapy, ferroptosis, breast cancer therapy

## Abstract

Developing clinically meaningful nanomedicines for cancer therapy requires the drugs to be effective, safe, simple, cheap, and easy to store. In the present work, we report that a simple cationic Fe(III)-rich salt of [Fe^III^Cl(TMPPH_2_)][Fe^III^Cl_4_]_2_ (**Fe-TMPP**) exhibits a superior anticancer performance on a broad spectrum of cancer cell lines, including breast, colorectal cancer, liver, pancreatic, prostate, and gastric cancers, with half maximal inhibitory concentration (IC_50_) values in the range of 0.098–3.97 μM (0.066–2.68 μg mL^−1^), comparable to the best-reported medicines. **Fe-TMPP** can form stand-alone nanoparticles in water without the need for extra surface modification or organic-solvent-assisted antisolvent precipitation. Critically, **Fe-TMPP** is TME-responsive (TME = tumor microenvironment), and can only elicit its function in the TME with overexpressed H_2_O_2_, converting H_2_O_2_ to the cytotoxic •OH to oxidize the phospholipid of the cancer cell membrane, causing ferroptosis, a programmed cell death process of cancer cells.

## 1. Introduction

**Scheme 1:**
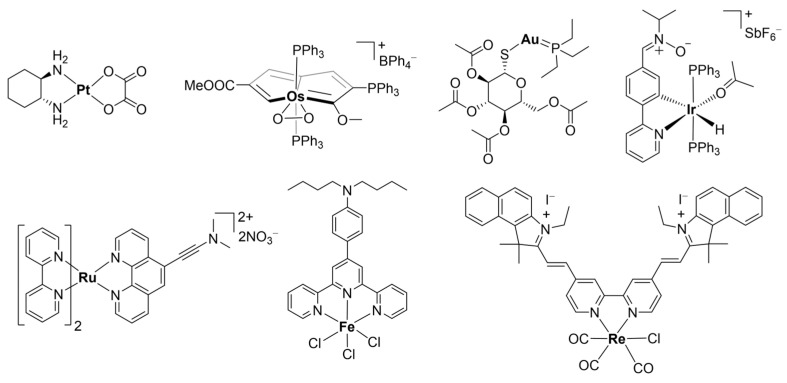
The structures of some reported ferroptosis inducers.

Materials designed to target the unique features of the tumor microenvironment (TME), such as the over-expression of H_2_O_2_ and glutathione (GSH), weak acidity, hypoxia, and immunosuppression, are attracting intensive interest in the field of nanomedicine [1,2,3,4,5,6]. Materials that are responsive exclusively to the TME imply low systemic toxicity and thus fewer side effects. Disturbing the complex homeostasis of the TME can further result in effective cancer therapy by novel cell death modalities (e.g., ferroptosis, pyroptosis, and immunogenic cell death) [7,8,9], and the resensitization of cells to conventional therapies [2,9,10]. Molecular complexes of Pt [11], Au [12], Fe [13], Os [14], Re [15], Ru [16], and Ir [17] have been reported to induce ferroptosis; their molecular structures are shown in Scheme 1. Nevertheless, most of the current formulations are far from satisfactory for practical use due to their formidable structural complexity, high cost, and low cellular uptake that subsequently leads to low anticancer efficacy.

Some useful strategies have been proposed to promote cellular uptake and the accumulation of the drugs in organelles (particularly mitochondria) [18,19] and the synergistic operation between different therapeutic modalities, such as chemotherapy, chemodynamic therapy (CDT), and photodynamic therapy (PDT), by taking advantage of the chemical properties of the individual building blocks of nanomedicines. For example, Ke et al. designed a cyclometallated Ir(III) complex featuring a −SH terminal as the cationic and photoactive building block [20]. Upon polymerization by disulfide bond formation, followed by DSPE–PEG–Biotin coating to endow aqueous solubility and cell specificity, the particle (denoted as IrS NPs) could be used for TME-responsive cancer therapy by harnessing both apoptosis and ferroptosis pathways. When IrS NPs were taken up by the cells, the disulfide bond was cleavaged by cross-coupling with the intracellular GSH to release the cationic subcomponent, which in turn, selectively accumulated in mitochondria. Notably, the use of Ir(III)-based photosensitizers elicited type I PDT, which circumvented the need for molecular oxygen. The oxidative stress imposed by the generated reactive oxygen species (ROS) from PDT resulted in the apoptosis of the lung cancer A549 cell line, while the depletion of GSH by IrS NPs weakened the reductive defense of the cells, leading to the effective peroxidation of the phospholipid, causing ferroptosis. In a similar effort, Yuan et al. designed a small molecular drug, MitoIrL2, featuring a cyclometallated Ir(III) skeleton for the hybrid treatment of breast and pancreatic cancer via a shared mechanism (apoptosis from type I PDT and ferroptosis) [21]. In MitoIrL2, the presence of an additional cationic phosphonium center resulted in more efficient mitochondrial accumulation and, therefore, better treatment outcomes relative to its phosphonium-free counterpart.

In the present work, we report that a simple salt of [Fe^III^Cl(TMPPH_2_)][Fe^III^Cl_4_]_2_ (denoted as **Fe-TMPP**; TMPPH_2_ is used to differentiate the ligand from the free-base porphyrin precursor of *meso*-Tetrakis (6-methylpyridin-3-yl) porphyrin, H_2_TMPP), which can directly form nanoparticles of suitable size for cellular uptake, and exhibits superior cytotoxicity towards a broad spectrum of cancer cell lines, including 4T1 (breast cancer), HCT-116 (colorectal cancer), DLD-1 (colorectal cancer), HuH-7 (liver cancer), BXPC3 (pancreatic cancer), PC3 (prostate cancer), and AGS (gastric cancer), with half maximal inhibitory concentration (IC_50_) values in the range 0.098−3.97 μM (0.066−2.68 μg mL^−1^), comparable to the best-reported medicines, including cisplatin and those exhibiting multiple treatment modalities.

Iron(II)-/Iron(III)- based nanoformulations have been widely reported for anticancer application via catalytic Fenton chemistry to convert the overexpressed H_2_O_2_ in TME into cytotoxic hydroxyl radicals (•OH) [6,22,23]. In addition, Fe^3+^ can be further reduced by the excess intracellular GSH into Fe^2+^ to initiate the Fenton process, and the exhaustion of GSH may further facilitate the ferroptosis process [6,22,23]. **Fe-TMPP**, reported herein, offers some advantages, such as iron-based chemotherapy, including inexpensive and facile synthesis, and direct nanoparticle formation without the need for complicated and costly surface modification. This instant nanoparticle formation by adding **Fe-TMPP** to water also permits convenient storage and simplifies the nanomedicine preparation process.

## 2. Results and Discussion

### 2.1. Material Synthesis and Structure Descriptions

**Figure 1:**
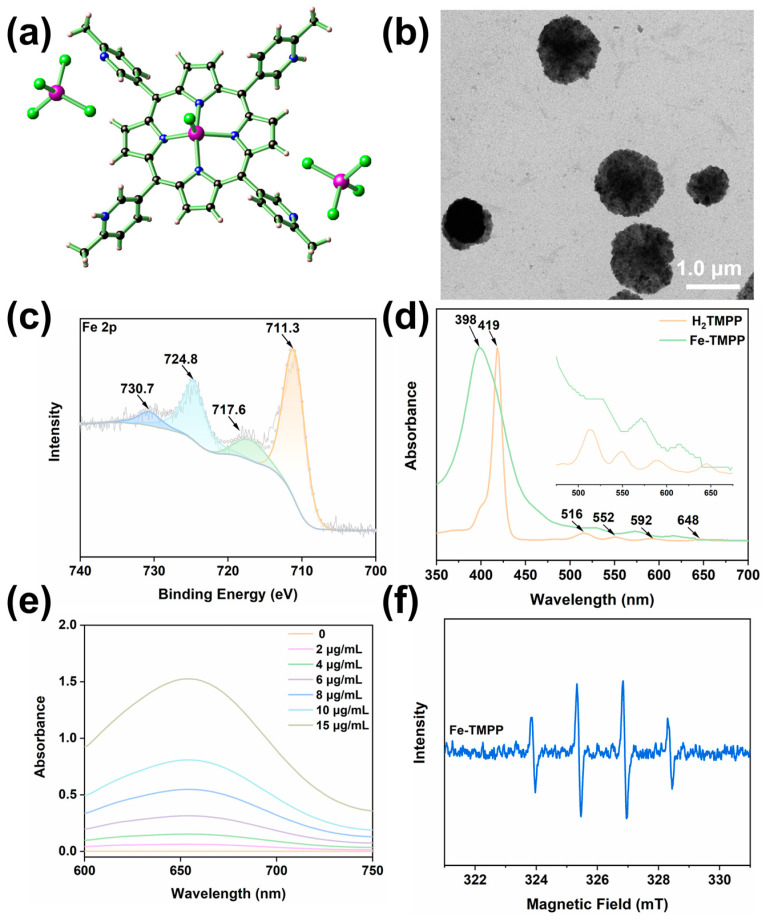
The crystal structure of **Fe-TMPP** with the disordered domain omitted for clarity ((**a**); Fe dark magenta, Cl green, N blue, C black, H light pink). TEM image (**b**) and the Fe 2p XPS spectra (**c**) of **Fe-TMPP**. A comparison of the Ultraviolet-visible (UV-Vis) spectra of H_2_TMPP (in N,N-dimethylformamide) and **Fe-TMPP** (in water) ((**d**); inset: the absorption in the range of 475−675 nm as amplified 10 times). Change of UV-Vis absorption intensity at 652 nm (oxTMB) upon treatment with different concentrations of **Fe-TMPP** in the presence of 100 μM H_2_O_2_ (**e**). Electron paramagnetic resonance (EPR) spectrum showing •OH production from H_2_O_2_ as induced by **Fe-TMPP** with 5,5-dimethyl-1-pyrroline-N-oxide (DMPO) as the spin trap (**f**).

The assembly of FeCl_3_·6H_2_O and H_2_TMPP under solvothermal conditions in CHCl_3_/EtOH using HAc as the modulating reagent resulted in the formation of **Fe-TMPP**. The successful synthesis was authenticated by single-crystal X-ray crystallographic analysis. **Fe-TMPP** crystallizes in the triclinic space group *P*−1 (Table 1), featuring a cation of [Fe^III^Cl(TMPPH_2_)]^2+^ and a pair of [FeCl_4_]^−^ counterions (Figure 1a). The [Fe^III^Cl(TMPPH_2_)]^2+^ cation features a ‘sit-atop’ Fe^3+^ which is chelated by four of the N atoms from the H_2_TMPP ligand and one Cl^−^ to give a five-coordinate square pyramidal configuration (Figure S1). Notably, of the four methylpyridyl peripheries of the H_2_TMPP ligand, the two N at the para-positions are protonated, as suggested by the presence of two [FeCl_4_]^−^ ions for each [Fe^III^Cl(TMPPH_2_)]^2+^ cation, in addition to the relative dispositions of these methylpyridyl moieties in the molecular packing diagram (Figure S2). The structure, refined with full protonation of the methylpyridyl N sites or misplaced protons, inevitably led to short H∙∙∙H contacts.

### 2.2. Spectroscopic Characterizations of Fe-TMPP

**Fe-TMPP** was spectroscopically characterized and compared with its H_2_TMPP precursor. The in-plane N-H vibration at 964 cm^−1^ in the Fourier transform infrared (FT-IR) spectrum of the ligand disappeared (Figure S3), and a new peak at 997 cm^−1^ appeared in **Fe-TMPP**, indicating the metalation of the porphyrin center. Fe 2p X-ray photoelectron spectroscopy (XPS) of **Fe-TMPP** revealed two peaks at binding energies of 711.3 eV and 724.8 eV, which are assignable by the spin-orbit splitting of the Fe 2p_3/2_ and Fe 2p_1/2_ of Fe^3+^ (Figure 1c), while a pair of satellite peaks at around 717.6 eV and 730.7 eV were also present, presumably due to the photoreduction of Fe^3+^ during the measurement [24,25].

In the ultraviolet–visible (UV-Vis) spectra (Figure 1d), the Soret band of the free H_2_TMPP at 419 nm underwent a significant blueshift to 398 nm upon the chelation of Fe^3+^. In addition, the Q bands coalesced into two, due to the change of symmetry from D_2h_ of H_2_TMPP to C_4v_ of Fe-TMPP [26,27]. Notably, an additional absorption band at around 650 nm also appeared, presumably due to the charge transfer between Fe^3+^ and the ligand [28]. Notably, the UV-Vis spectrum of **Fe-TMPP** exhibited an undulating baseline, indicating lighting scattering by nanoparticle formation in an aqueous solution [29]. The formation of a stand-alone nanoparticle of **Fe-TMPP** could be directly observed with the Tyndall effect (Figure S4). Energy dispersive X-ray spectroscopy (EDS) showed an Fe:Cl:N atomic ratio of 1.0:2.6:3.0 in **Fe-TMPP** (Figure S5a), which is close to the value of 1.0:3.0:2.7 found in the single crystal data.

### 2.3. Characterizations of Fe-TMPP Nanoparticles

The direct nanoparticle formation of **Fe-TMPP** in water was observed in the transmission electron microscopic (TEM) images and also by dynamic light scattering (DLS) analysis. TEM showed that **Fe-TMPP** formed particles of variable sizes (Figure 1b). DLS gave different results from batch to batch but all contained considerable numbers of particles with sizes less than 100 nm (Figure S6). We argue that the accuracy of the TEM results may be compromised, as the particles of **Fe-TMPP**, without any surface modification, may undergo an Ostwald ripening process upon solvent evaporation during sample preparation [30]. The zeta potential of **Fe-TMPP** was 23.4 mV (Figure S7), which is ideal for ensuring good circulation stability with favorable adhesion to cell membranes for enhanced intracellular uptake [31].

Nanoparticles can be assembled and stored in an aqueous solution for short periods, or lyophilized for longer-term preservation. However, lyophilized particles can change their morphology, aggregate, or slowly degrade [32,33]. Antisolvent precipitation is an often-used alternative to prepare nanoparticles of organic drugs. A risk with this approach, however, is the encapsulation of toxic organic solvents, and sometimes the need for a supercritical antisolvent (e.g., CO_2_) [34,35]. The simple chemical formulation and good water-dispersing ability of **Fe-TMPP** is thus an advantage for storage and facile particle preparation.

Fresh samples of **Fe-TMPP** in aqueous solutions maintained their color and homogeneity under ambient conditions for three days (Figure S8), as also evidenced by their UV-Vis spectra (Figure S9). In addition, **Fe-TMPP** was stable in PBS (0.1×) for 0, 12, and 24 h, as indicated by only insignificant changes in particle size (Figure S10) and zeta potential (Figure S11). To further confirm the stability of **Fe-TMPP** in water, we sonicated the aqueous suspensions of **Fe-TMPP** and then lyophilized. The powder X-ray diffraction (PXRD) analysis revealed that the diffraction patterns of the water-treated samples resembled that of the as-synthesized crystalline samples (Figure S12). Moreover, the EDS spectra of the lyophilized sample of **Fe-TMPP** showed an Fe:Cl:N atomic ratio of 1.0:2.4:3.2 (Figure S5b), which was close to that of the as-synthesized crystalline sample (1.0:2.6:3.0), indicating that there was no obvious compositional change upon suspending **Fe-TMPP** in water. We postulate that the four methyl groups at the ortho position of the pyridyl N sites significantly boost the hydrophobicity of the molecule to facilitate the aggregation of the polar **Fe-TMPP** featuring multiple hydrophilic moieties (i.e., [FeCl_4_]^−^, protonated pyridyl ligands, the Fe-Cl, and free pyridyl N site) and thereby protecting the salt from hydrolyzation.

### 2.4. Detection of Fe-TMPP-induced Generation of ROS in Solution

High valent transition metal ions Fe^3+^ and Cu^2+^ exhibit peroxidase-like activity to generate reactive •OH, by utilizing the overexpressed H_2_O_2_ in the TME, thereby eliciting nonspecific damage inside cancer cells [6,7,8,26]. The TME also reduces Fe^3+^ and Cu^2+^ to Fe^2+^ and Cu^+^ through a reaction with the overexpressed GSH to initiate Fenton and Fenton-like reactions, which also generate •OH from H_2_O_2_. The peroxidase-like activity of the Fe^3+^-rich **Fe-TMPP** was therefore assayed using 3,3′,5,5′-tetramethylbenzidine (TMB) [36]. Different concentrations of **Fe-TMPP** were added to TMB solutions containing 100 μM H_2_O_2_, the approximate concentration in tumors, and five times higher than in normal cells [1,2]. It can be seen from Figure 1e that **Fe-TMPP** produced considerable •OH in a concentration-dependent manner upon incubating with H_2_O_2_ over 3 h. The generation of •OH was confirmed by electron paramagnetic resonance (EPR) spectroscopy, using DMPO as the spin trap. As shown in Figure 1f, the EPR spectrum of **Fe-TMPP** gave rise to a strong four-line signal with a 1:2:2:1 peak-to-peak intensity pattern, characteristic of the generation of •OH from H_2_O_2_ [4,5].

**Fe-TMPP** is supported by a porphyrin-based ligand, which is often used for type II PDT of cancer [3,7,37]. We employed diphenylisobenzofuran (DPBF) as a probe to compare the singlet oxygen’s (^1^O_2_) generating capacity with that of **TMPP-F127** (H_2_TMPP encapsuled by Pluronic F-127, Figure S13) [38], using blank DPBF as a control [39,40]. DPBF was added to aqueous solutions of **TMPP-F127** and **Fe-TMPP**, followed by laser irradiation (650 nm, 25 mW cm^−2^). As shown in Figure S14, at equivalent H_2_TMPP concentrations, neither species could generate sufficient ^1^O_2_ to degrade DPBF under laser irradiation. The ^1^O_2_ generated by **Fe-TMPP** was negligible, which might be due to the presence of redox-active Fe^3+^ that can quench the excited state of the molecule by electron transfer [41,42].

### 2.5. In Vitro ROS Detection and Cytotoxicity Assay

**Figure 2:**
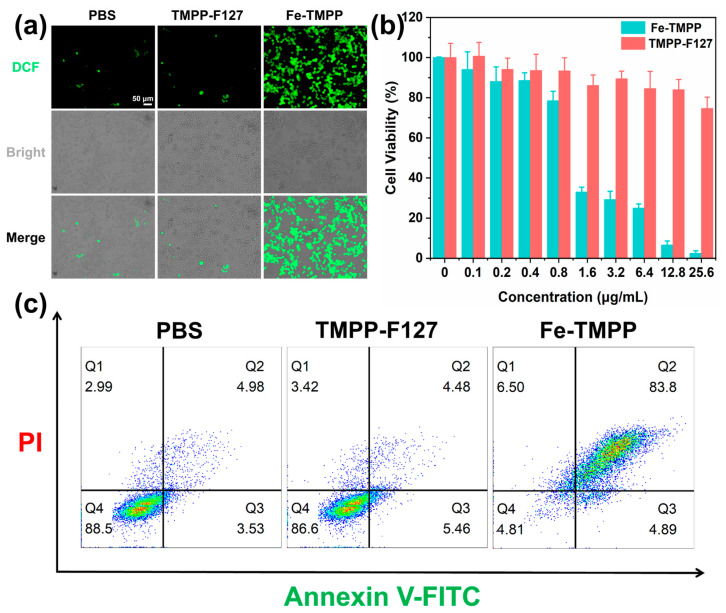
The generation of intracellular ROS by **TMPP-F127**, **Fe-TMPP**, and PBS control, as measured by DCF fluorescence (**a**). A comparison of the 4T1 cell viability when treated with gradient concentrations of **TMPP-F127** and **Fe-TMPP** (**b**). Flow cytometric evaluation of 4T1 cell apoptosis by **Fe-TMPP**, **TMPP-F127**, and a PBS using the Annexin V-PI double staining method (**c**).

Given the efficient •OH production in water induced by **Fe-TMPP**, we next investigated in vitro ROS generation by **Fe-TMPP** in the representative 4T1 cell line using 2′,7′-dichlorodihydrofluorescein diacetate (DCFH-DA) as a fluorescence probe [4]. As shown in Figure 2a and Figure S15, green fluorescence was detected in the cells treated with **Fe-TMPP**, which indicated the production of ROS in the cells from the peroxidase-like activity of Fe^3+^. By comparison, **TMPP-F127** produced few ROS, which may be due to the aggregation of the H_2_TMPP ligand within the hydrophobic core of the **TMPP-F127** micelles.

The cytotoxicity of **Fe-TMPP** against diverse cell lines including 4T1 (breast cancer), HCT-116 (colorectal cancer), DLD-1 (colorectal cancer), HuH-7 (liver cancer), BXPC3 (pancreatic cancer), PC3 (prostate cancer), and AGS (gastric cancer), were evaluated using either 3-(4,5-dimethylthiazol-2-yl)-2,5-diphenyltetrazolium bromide (MTT) or cell counting kit-8 (CCK-8) assays. As shown in Figure 2b and Figure S16, **Fe-TMPP** exhibited concentration-dependent cytotoxicity against all tested cell lines. The half maximal inhibitory concentration (IC_50_) values for **Fe-TMPP** were relatively low, down to 0.0975 μM (0.0658 μg mL^−1^) for AGS and up to 3.97 μM (2.68 μg mL^−1^) for HCT-116 (Table 2). These IC_50_ values are comparable to cisplatin and to complex formulations that integrate multiple therapeutic modalities. The above results indicate that **Fe-TMPP** exhibits significant cytotoxicity against a broad spectrum of cancer cell lines.

### 2.6. Ferroptosis Assay

**Figure 3:**
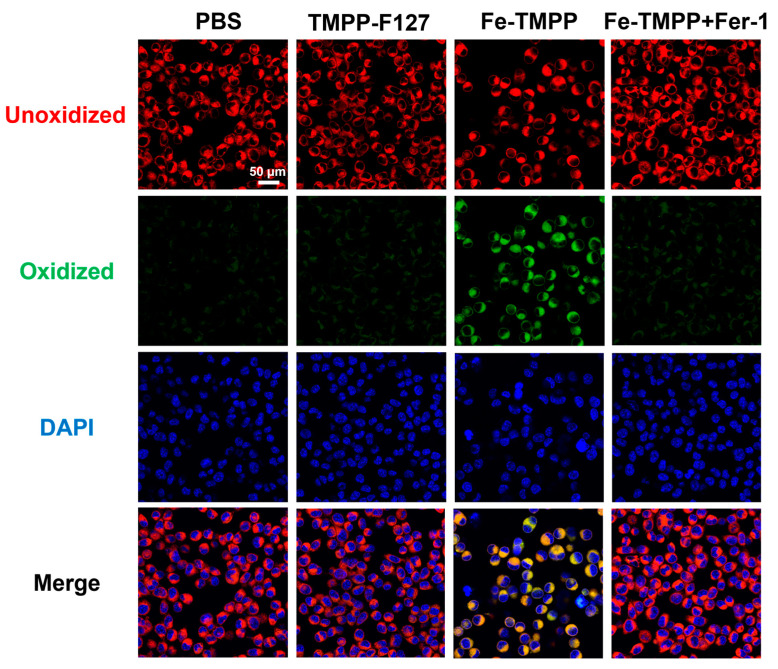
The confocal laser scanning microscopic images of the accumulation of lipid peroxidation in 4T1 cells when treated with PBS control, **TMPP-F127**, **Fe-TMPP**, and **Fe-TMPP** in the presence of Fer-1 as the ferroptosis inhibitor using C11- BODIPY as the fluorescent probe.

As noted above, when **Fe-TMPP** is internalized by cancer cells, Fe^3+^ can oxidize GSH to give glutathione disulfide (GSSG) and is concomitantly reduced to Fe^2+^. The exhaustion of GSH in these cells can downregulate the expression of glutathione peroxidase 4 (GPX4), making the cell membrane phospholipid more susceptible to peroxidation [49,50]. GSH depletion and phospholipid peroxidation are two characteristic features of ferroptosis, a novel form of iron-dependent programmed cell death [49,50]. We therefore examined the possibility of ferroptosis induced by **Fe-TMPP** using 4T1 cell line as a representative. Phospholipid peroxidation levels of 4T1 cells were measured using C11-BODIPY (BODIPY™ 581/591 C11) as a fluorescent probe, as observed under confocal laser scanning microscopy (Figure 3) [51]. We also used DAPI to stain the nucleus of the cells. The cells treated with **Fe-TMPP** showed significantly stronger lipid peroxidation than those treated with **TMPP-F127** or PBS. In addition, lipid peroxidation can be inhibited by the presence of ROS scavengers, such as ferrostatin-1 (Fer-1), liproxstatin-1, and vitamin E [21,52]. In the present experiments, lipid peroxidation was completely inhibited by the introduction of Fer-1, as indicated by the restoration of the red emission of C11-BODIPY. These results indicated that **Fe-TMPP** can induce ferroptosis for cancer treatment. Flow cytometry using Annexin-V/PI double staining further confirmed that 4T1 cells treated with **Fe-TMPP** showed obviously late apoptosis relative to **TMPP-F127** and PBS-treated cells, supporting this alternative cell death mechanism (Figure 2c) [53,54].

## 3. Materials and Methods

### 3.1. General

Ligand H_2_TMPP was synthesized as described in our previous reports [27]. 3-Aldehyde-6-methylpyridine (>98%, Macklin, Shanghai, China), ferric chloride hexahydrate (99%, Macklin), pluronic F-127 (average molecular weight 2000, Sigma-Aldrich, Darmstadt, Germany), 1,3-diphenylisobenzofuran (DPBF) (97%, Acros, Waltham, MA, USA), 3,3′,5,5′-tetramethylbenzidine (TMB) (99%, Shanghai Yuanye Bio-Technology Co., Ltd., Shanghai, China), and hydrogen peroxide (H_2_O_2_, 30%) (Yikewei Biological Technology Co., Ltd., Guangzhou, China) were obtained directly from commercial sources and used as received. Pyrrole (>95%, Macklin) was freshly distilled before use.

HCT-116, HuH-7, BXPC3, DLD-1, PC3, AGS, and 4T1 cell lines were purchased from the Shanghai Institute of Cell Biology, Chinese Academy of Sciences. Phosphate buffer solution (PBS) and fetal bovine serum (FBS) supplemented with penicillin and streptomycin were bought from Elabscience Crop (Guangzhou, China). 3-(4,5-Dimethylthiazol-2-yl)-2,5-diphenyltetrazolium bromide (MTT) was purchased from Beijing Solarbio Science & Technology Co., Ltd. (Beijing, China). The cell counting kit-8 (CCK-8) was commercially available from APEXBIO. 2′,7′-Dichlorodihydrofluorescein diacetate (DCFH-DA) and 4′,6-diamidino-2-phenylindole (DAPI) solution were obtained from Beijing Solarbio Science & Technology Co., Ltd. C11-BODIPY^581/591^ was bought from Invitrogen Corp. Ferrostatin-1 (Fer-1) was bought from Yike Biological Technology Co., Ltd. (Guangzhou, China). The Annexin FITC/PI apoptosis detection kit was purchased from Elabscience Crop.

Fourier transform infrared (FT-IR) spectra were recorded on a Bruker VERTEX 70+HYPERION 2000 spectrometer. Ultraviolet-visible spectroscopy (UV-Vis) absorption spectra were obtained on a Varian Cary-50 UV–Visible spectrophotometer (Varian, Inc., Palo Alto, CA, USA). X-ray photoelectron spectroscopy (XPS) was conducted on an EXCALAB 250 XI X-ray photoelectron spectrometer (Thermo Scientific, Waltham, MA, USA). The EPR measurement was conducted by a Bruker EMXnano spectrometer, with DMPO as the spin-trapping agent. The optical density (OD) values were collected using a TECAN M1000PRO microplate reader (Tecan, Zürich, Switzerland). The flow cytometry was performed using a BD FACS Caliber instrument (BD Biosciences, San Jose, CA, USA). The transmission electron microscopic (TEM) images were obtained by dropping the sample in water onto a copper net under a HITACHI HT7700 transmission electron microscope. Zeta potentials were measured on an LA-95052 laser particle size analyzer using dynamic light scattering (DLS). The cytotoxicity assay was conducted on a multifunction microplate detector by recording the absorption at 570 nm using a TECAN M1000PRO microplate reader. The cell staining results were observed with a confocal laser scanning microscope (CLSM, AxioObserver A1, Germany). The EDS and elemental mapping diagrams were obtained on the EVO18 scanning electron microscope.

### 3.2. Synthesis and Characterization of TMPP-F127

Pluronic F-127 (10.6 mg) was introduced to 1 mL of DMSO solution containing H_2_TMPP (4.5 mg), and the homogenous mixture formed was then added dropwise into 3.5 mL of deionized water with tip sonication. The micelle formed was dialyzed using a dialysis bag (molecular weight cut-off of 2000 Da) for 24 h to obtain **TMPP-F127**. TEM indicated that the particle sizes for **TMPP-F127** were 170 ± 25 nm (Figure S13), which is conducive to cellular uptake.

### 3.3. Synthesis and Characterization of Fe-TMPP

FeCl_3_·6H_2_O (2.4 mg, 0.009 mmol) and H_2_TMPP (2 mg, 0.003 mmol) in 2 mL of CHCl_3_/EtOH (v:v = 3:1) were added into a Pyrex glass tube, followed by HAc (50 μL) as a regulator. The resulting mixture was then transferred to a programmable oven, heated to 120 °C over 4 h, and maintained at that temperature for 48 h, before cooling to room temperature over 24 h to yield purple block crystals of **Fe-TMPP**, which were collected through filtration, washed thoroughly with anhydrous ether, and dried in vacuo. Yield: 0.84 mg, 24.2% based on Fe. Anal. Calcd (%) for C_44_H_34_Cl_9_Fe_3_N_8_: C 45.50%, H 2.95%, N 9.65%; found: C 41.87%, H 2.74%, and N 8.70%, corresponding to **Fe-TMPP**∙CHCl_3_ (C 42.20%, H 2.75%, and N 8.75%). FT-IR (KBr disc, cm^−1^): 3372(w), 2521(w), 2162(w), 2029(w), 1607(m), 1552(m), 1458(m), 1377(w), 1329(w), 1288(s), 1258(w), 1204(m), 1139(s), 1085(m), 1047(m), 997(vs), 890(w), 854(w), 801(vs), 744(s), 721(s), 662(m), and 624(w).

### 3.4. Single Crystal X-ray Crystallography

Data collections were performed on a Bruker APEX II CCD X-ray diffractometer using Mo Kα (λ = 0.71073 Å) irradiation. Refinement and reduction of the collected data were achieved using the program Bruker SAINT and absorption corrections were performed using a multi-scan method [55]. Crystal structures were solved by direct methods and refined on F^2^ using full-matrix least-squares techniques with *SHELXTL-2016* [56].

For **Fe-TMPP**, the [Fe^III^Cl_4_]^−^ anion displays a conformational disorder with a relative ratio of 0.73:0.27 refined for the disordered domains. The [Fe^III^Cl(TMPPH_2_)]^2+^ cation also has two conformations due to the presence of ‘sit-atop’ Fe^3+^, and the disorder is at about the center of the porphyrin ligand. Thus, the occupancy factor of the coordinated FeCl moiety was fixed at 0.5. For the porphyrin ligand, only two pyridyl groups were protonated to balance the charge of the anion. The protonation of half of the four pyridyls can also be judged from the packing of the molecules. In the packing diagram, two pyridyls were brought to proximity by an N-H∙∙∙N bond, thereby excluding the full protonation of the pyridyls and corroborating the charge counting. A small amount of spatially delocalized electron density in the lattice was found but acceptable refinement results could not be obtained for this electron density. The solvent contribution was then modeled using SQUEEZE in the Platon program suite [57].

Crystallographic data for **Fe-TMPP** have been deposited in the Cambridge Crystallographic Data Center (CCDC) as supplementary publication number 2330690. These data can be obtained free of charge from the CCDC via www.ccdc.cam.ac.uk/data_request/cif accessed on 3 February 2024) or from the Supplementary Materials. A summary of the key crystallographic data for **Fe-TMPP** is listed in Table 1.

### 3.5. •OH Detection

Different concentrations of **Fe-TMPP** were added to a TMB solution containing 100 μM H_2_O_2_. After reacting for 3 h at room temperature, the solution was centrifuged to remove the influence of nanoparticles on absorbance. The ultraviolet absorption curve of the supernatant at 652 nm was determined.

### 3.6. ^1^O_2_ Detection

Identical concentrations of DPBF were added to aqueous solutions of **TMPP-F127** and **Fe-TMPP** relative to the concentration of TMPP, to give uniform mixtures, with the concentrations of DPBF and TMPP being 33 μg mL^−1^ and 7 μg mL^−1^, respectively. The solutions were then irradiated by a laser (650 nm, 25 mW cm^−2^). After each irradiation for 30 s, the absorption of DPBF was immediately measured. By monitoring the decreasing rate of the ultraviolet absorption peak at 416 nm, the ^1^O_2_ generation capacities of these materials could be evaluated.

### 3.7. MTT or CCK-8 Cytotoxicity Assay

The MTT assay of 4T1 cells is detailed as an example. The 4T1 cell line was cultured in Roswell Park Memorial Institute (RPMI) 1640 media supplemented with 10% FBS and 1% penicillin-streptomycin at 37 °C in 5% CO_2_. Cells grew as a monolayer and were detached upon confluence using trypsin (0.5% *w*/*v* in PBS). The cells were harvested from the cell culture medium by incubating in trypsin solution for 2 min, and then centrifuged with the supernatant subsequently discarded. A 3 mL portion of serum-supplemented cell culture medium was added to neutralize any residual trypsin. The cells were re-suspended in serum-supplemented RPMI 1640 at a concentration of 5 × 10^4^ cells/mL. Cells were cultured at 37 °C and 5% CO_2_ for the MTT studies.

4T1 cells were inoculated into 96-well plates (5 × 10^3^ cells per well) and incubated for 24 h. After 24 h of incubation, the cells were treated with different concentrations of drugs for 24 h, and, upon completion of the treatment, the MTT solution was added and incubated for 4 h, and DMSO was added and shocked for 15 min. The cell viability was determined using an enzyme marker.

The relative cell viability (%) related to control cells was calculated using the following equation:

$$V\%=\frac{{\left[A\right]}_{experimental}-{\left[A\right]}_{blank}}{{\left[A\right]}_{control}-{\left[A\right]}_{blank}}\times 100\%$$

where *V*% is the percentage of cell viability, [A]*_experimental_* is the absorbance of the wells culturing the treated cells, [A]*_blank_* is the absorbance of the blank, and [A]*_control_* is the absorbance of the wells culturing untreated cells.

The cell viability data for HCT-116 (CCK-8), DLD-1 (CCK-8), HuH-7 (CCK-8), BXPC3 (CCK-8), PC3 (CCK-8), and AGS (CCK-8) were obtained in a similar manner using protocols listed in Table S1.

### 3.8. Intracellular ROS Detection

4T1 cells were inoculated in 12-well plates (1 × 10^5^ cells per well), incubated for 24 h and then treated with different drugs for 24 h. After completion of the treatment, the cells were treated with DCFH-DA, and the cellular reactive oxygen species results were determined using an inverted fluorescence microscopy.

### 3.9. Flow Cytometric Apoptosis Assay

4T1 cells were inoculated in 6-well plates (3 × 10^5^ cells per well) and incubated for 24 h. After 24 h of incubation, the cells were treated with different drugs for 24 h. After the completion of the treatment, the cells were processed using an Annexin V-FITC/PI apoptosis kit, and the results of apoptosis were determined using flow cytometry.

### 3.10. Ferroptosis Assay

4T1 cells were inoculated in confocal glass dishes (1 × 10^5^ cells per dish) and incubated for 24 h. Fer-1 (10 μM), an inhibitor of ferroptosis, was added and incubated for 12 h. The cells were then treated with different drugs for 24 h. After the completion of the treatment, the cells were incubated for 30 min with the ferroptosis probe C11-BODIPY^581/591^ (10 μM) and the results of the ferroptosis were observed using CLSM.

## 4. Conclusions

In this work, a simple ferroptosis inducer of **Fe-TMPP** was readily synthesized from earth-abundant metal and common organic materials. **Fe-TMPP** can directly form nanoparticles without requiring extra surface modification or organic-solvent-assisted antisolvent precipitation. Compared to the reported ferroptosis inducers based on noble metals such as oxaliplatin [11] and other coordination complexes derived from Au [12], Os [14], Ru [16], and Ir [17], the advantages of using **Fe-TMPP** include inexpensive fabrication, in addition to its cancer-specific activity and promising cytotoxicity, as described herein. The instant nanoparticle formation by adding **Fe-TMPP** to water also ensures convenient storage and simplifies the potential nanomedicine preparation process. All these features are important for potential clinical use. However, it should also be noted that iron overloading can potentially lead to cardiotoxicity, also caused by the ferroptosis process [58,59]. Nevertheless, the unexpectedly high cytotoxicity originating from ROS alone inspires us to investigate the possibility of other structure analogs based on metal ions, such as Cu^2+^, and to further explore their mechanisms of action by examining the sub-organelle locations of these metallodrugs.

## Figures and Tables

**Table 1:** Crystal data and structure refinement parameters for **Fe-TMPP**.

	Fe-TMPP
CCDC number	2330690
Formula	C_44_H_34_Cl_9_Fe_3_N_8_
Formula weight	1161.39
Crystal system	Triclinic
Space group	*P* − 1
*T* (K)	213(2)
*a*/Å	9.5980(6)
*b*/Å	11.0408(7)
*c*/Å	14.4682(10)
*α*/°	70.702(3)
*β*/°	86.792(3)
*γ*/°	75.171(2)
*V*/Å^3^	1398.16(16)
*Z*	1
*D_c_*/(g cm^−3^)	1.379
*F*(000)	585
*μ* (Mo–Kα)/mm^−1^	1.234
Total reflections	29,635
Unique reflections	4006
Observed reflections	2844
No parameters	339
*R*_int_	0.0778
*R ^a^*	0.1007
*wR ^b^*	0.2811
*GOF ^c^*	1.055

^a^*R* = Σ||*F*_o_| − |*F*_c_||/Σ|*F*_o_|. ^b^ *wR* = {Σ*w*(*F*_o_^2^ − *F*_c_^2^)^2^/Σ*w*(*F*_o_^2^)^2^}^1/2^. ^c^ *GOF* = {Σ*w*((*F*_o_^2^ − *F*_c_^2^)^2^)]/(*n* − *p*)}^1/2^, where *n* = number of reflections and p = total number of parameters refined.

**Table 2:** A comparison of the IC_50_ values of cell lines induced by the reported drugs and **Fe-TMPP**.

Drug Formulation	Cell Line	IC_50_	Reference
Cisplatin	4T1	7.43 μM	[43]
Cisplatin	MCF7	43.0 μM	[44]
Cisplatin	A549	16.4 μM	[45]
Cisplatin	HT29	24.8 μM	[45]
Fe-TBP	CT26	3.10 μM	[46]
bcc-USINPs	Hep-G2	15.7 μg mL^−1^	[47]
AE@RBC/Fe NCs	HSC-3	7.80 μM	[48]
**Fe-TMPP**	HCT-116	3.97 μM	This work ^†^
**Fe-TMPP**	4T1	2.00 μM	This work ^†^
**Fe-TMPP**	HuH-7	3.68 μM	This work ^†^
**Fe-TMPP**	BXPC3	3.11 μM	This work ^†^
**Fe-TMPP**	DLD-1	0.231 μM	This work ^†^
**Fe-TMPP**	PC3	0.213 μM	This work ^†^
**Fe-TMPP**	AGS	0.0975 μM	This work ^†^

^†^ Corresponding IC_50_ values in μg mL^−1^: HCT-116 (2.68); 4T1 (1.35); HuH-7 (2.49); BXPC3 (2.10); DLD-1 (0.156); PC3 (0.144); AGS (0.0658).

## Data Availability

The data that support the findings of this study are available from the corresponding authors upon request.

## Supplementary Materials

The following supporting information can be downloaded at: https://www.mdpi.com/article/10.3390/molecules29112495/s1.
