# Supplementary material for: [FeIIICl(TMPPH2)][FeIIICl4]2: A Stand-Alone Molecular Nanomedicine That Induces High Cytotoxicity by Ferroptosis
Source: Molecules. 2024 May 24;29(11):2495. doi: 10.3390/molecules29112495 (PMC11173869; doi:10.3390/molecules29112495)
Supplement: Supplementary file 1 [file molecules-29-02495-s001.zip › molecules-3009966-supplementary.pdf]

Supplementary Material for

$[Fe^{III}Cl(TMPPH_2)][Fe^{III}Cl_4]_2$: A Stand-Alone Molecular Nanomedicine That Induces High Cytotoxicity by Ferroptosis

Xiao Wang [1,†], Jia-Hao Feng [2,†], Chun-Mei Zeng [1], Ze-Sheng Zhang [1], Feng-Lin Cao [1], Wen-Hua Zhang [1,*], Jin-Xiang Chen [2,*] and David J. Young [3]

[1] College of Chemistry, Chemical Engineering, and Materials Science, Soochow University, Suzhou 215123, China; wxxx0225@163.com (X.W.); 20214209084@stu.suda.edu.cn (C.-M.Z.); 20224209184@stu.suda.edu.cn (Z.-S.Z.); xingyue5435@163.com (F.-L.C.)

[2] NMPA Key Laboratory for Research and Evaluation of Drug Metabolism, Guangdong Provincial Key Laboratory of New Drug Screening, School of Pharmaceutical Sciences, Southern Medical University, Guangzhou 510515, China; 13078100247@163.com

[3] Glasgow College UESTC, University of Electronic Science and Technology of China, Chengdu 611731, China; david.j.young@glasgow.ac.uk

* Correspondence: whzhang@suda.edu.cn (W.-H.Z.); jxchen@smu.edu.cn (J.-X.C.)

† These authors contributed equally to this work.

Table of Contents

Figure S1. Top (a) and side (b) view of the $[Fe^{III}Cl(TMPPH_2)]^+$ cation in **Fe-TMPP**, showing a pair of 'sit-atop' FeCl units disordered over the porphyrin plane. Legend: Fe shiny and plain magenta, Cl shiny and plain green, N blue, C black, and H light pink.. 3

Figure S2. The molecular packing diagram of **Fe-TMPP** looking along the crystallographic *a* direction showing the H-bonding among the molecules.. 4

Figure S3. A comparison of the FT-IR spectra of H_2TMPP and **Fe-TMPP**.. 5

Figure S4. Tyndall effect of **Fe-TMPP** in the aqueous solution. 6

Figure S5. The EDS elemental ratios of the as-synthesized **Fe-TMPP** (a) and that from the lyophilized sample (b; 10 mg in 10 mL of water, sonicated and kept for 24 h), showing no obvious change of the chemical composition.. 7

Figure S6. The DLS results of three **Fe-TMPP** aqueous solutions. 8

Figure S7. The zeta potential results of **Fe-TMPP** in aqueous solution. 9

Figure S8. Pictures of **Fe-TMPP** dispersed in aqueous solution for 0–3 days.................................. 10

Figure S9. UV-Vis absorption spectra of **Fe-TMPP** dispersed in aqueous solution for 0–3 days....... 11

Figure S10. The DLS results of **Fe-TMPP** in PBS (0.1x) for 0 (a), 12 (b), and 24 hours (c). 12

Figure S11. The zeta potential results of **Fe-TMPP** in PBS (0.1x) for 0 (a), 12 (b) and 24 hours (c)..... 13

Figure S12. A comparison of the PXRD spectra of **Fe-TMPP** and its lyophilized samples after sonication and kept in different amounts of water for 24 h. 14

Figure S13. TEM image of **TMPP-F127**. 15

Figure S14. UV-Vis absorption spectra of DPBF (a) and its dispersion in **TMPP-F127** (b) and **Fe-TMPP** (c) aqueous solution with different illumination times. The changes of absorption intensity at 416 nm of DPBF as a function of irradiation time when incubated with **TMPP-F127** and **Fe-TMPP** using blank DPBF as a control (d). 16

Figure S15. A comparison of fluorescence intensity of DCF when treated with **TMPP-F127**, **Fe-TMPP**, and PBS control.. 17

Figure S16. A comparison of the HCT-116 (a), HuH-7 (b), and BXPC3 (c) cell viabilities when treated with gradient concentrations of **TMPP-F127** and **Fe-TMPP**. The DLD-1, PC3, and AGS cells viabilities when treated with gradient concentrations of **Fe-TMPP** (d)... 18

Table S1. Detailed cell development and drug incubation conditions.. 19

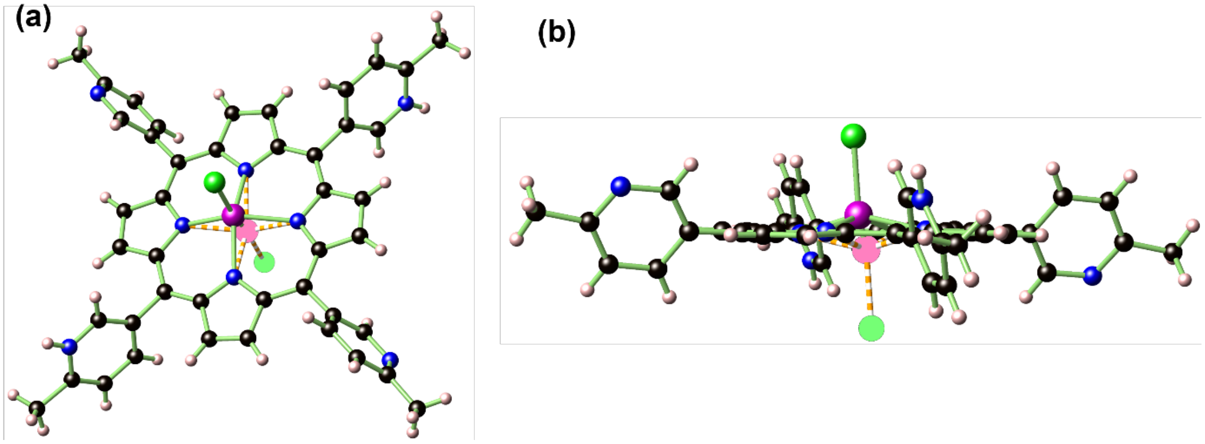

Figure S1. Top (a) and side (b) view of the [$Fe^{III}Cl(TMPPH_2)]^+$ cation in **Fe-TMPP**, showing a pair of 'sit-atop' FeCl units disordered over the porphyrin plane. Legend: Fe shiny and plain magenta, Cl shiny and plain green, N blue, C black, and H light pink.

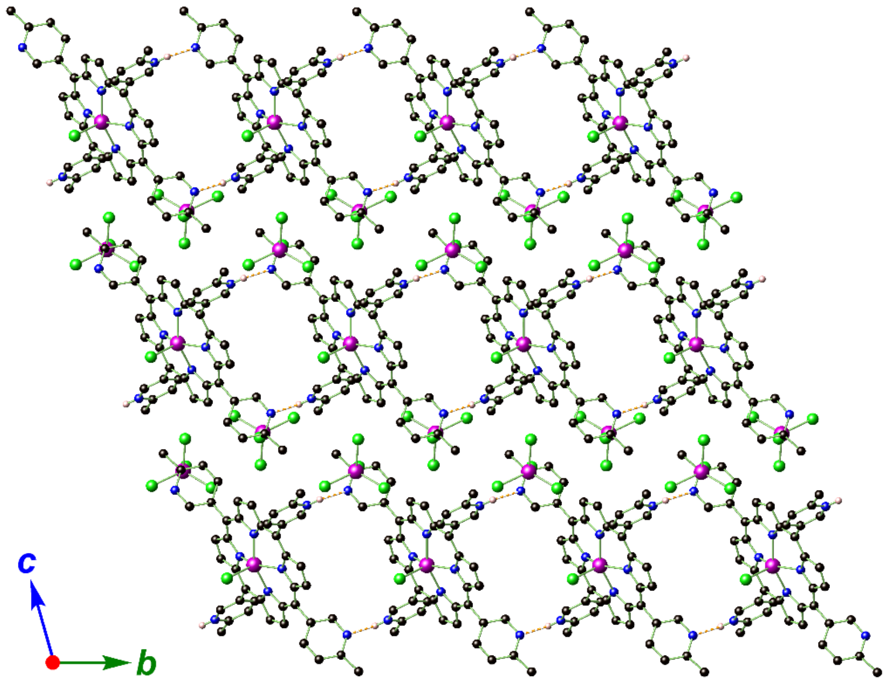

Figure S2. The molecular packing diagram of **Fe-TMPP** looking along the crystallographic *a* direction showing the H-bonding among the molecules.

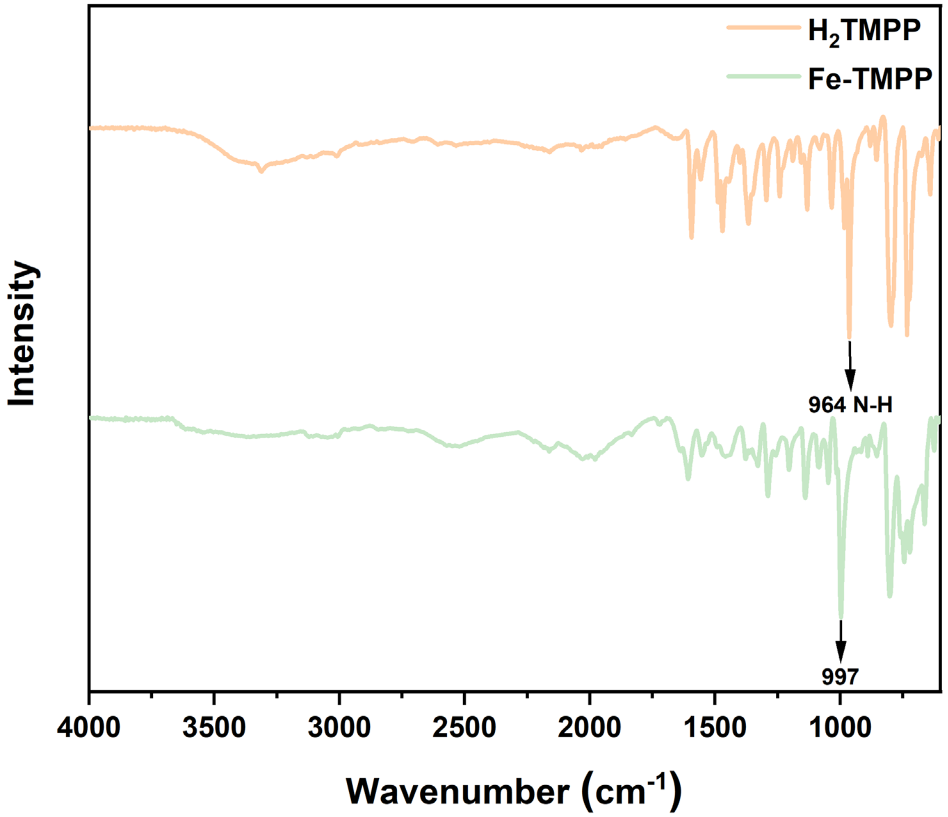

Figure S3. A comparison of the FT-IR spectra of H_2TMPP and **Fe-TMPP**.

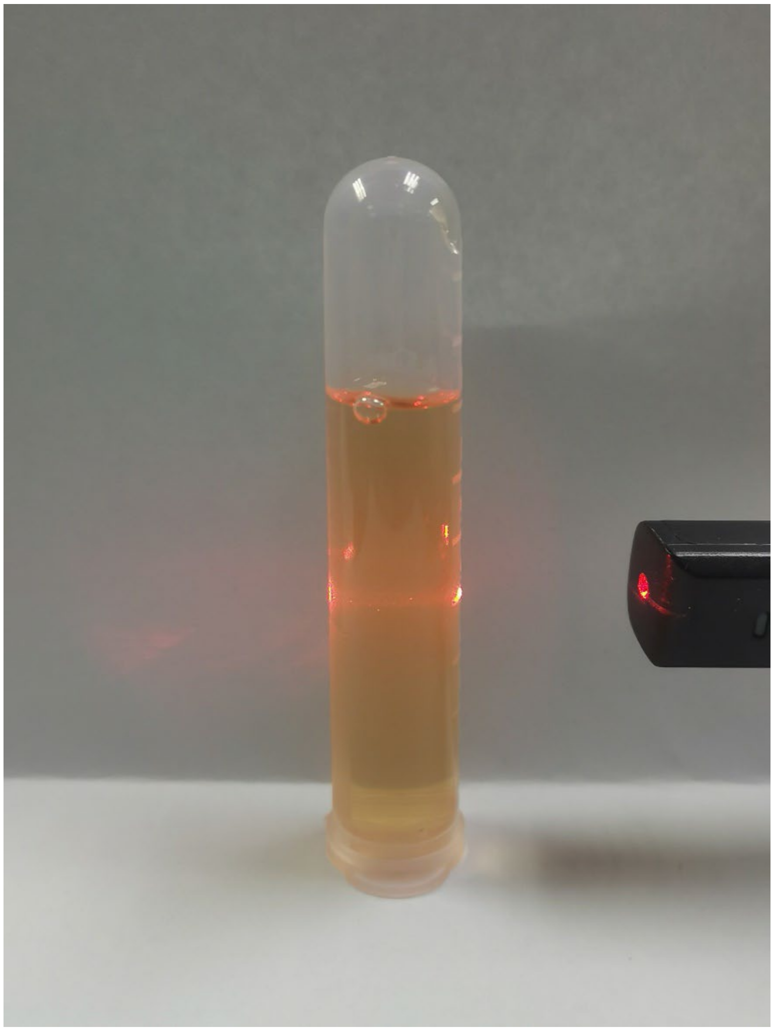

Figure S4. Tyndall effect of **Fe-TMPP** in the aqueous solution.

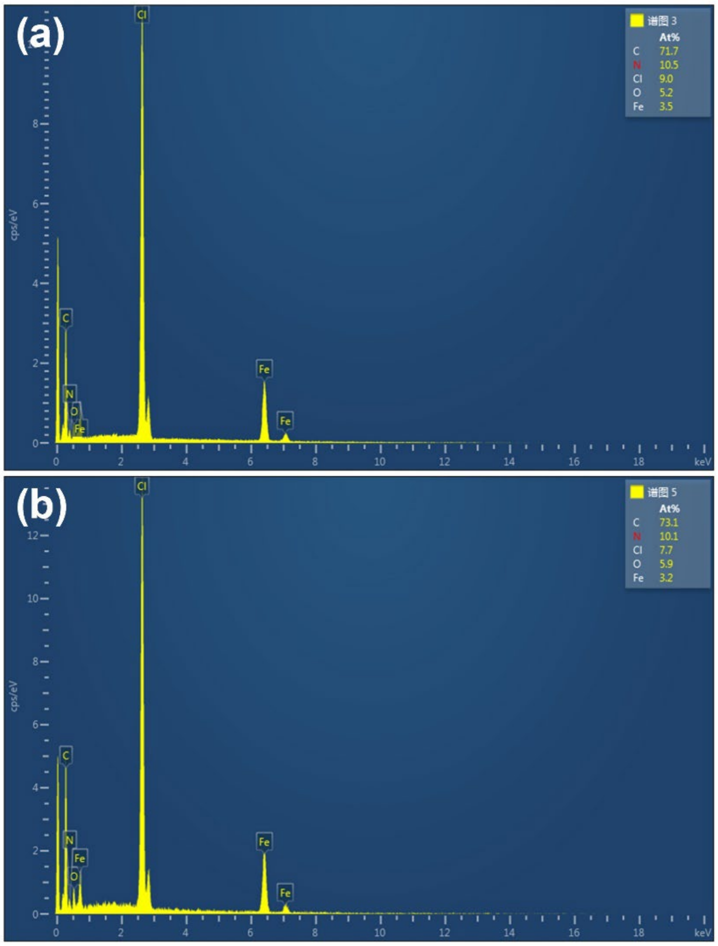

Figure S5. The EDS elemental ratios of the as-synthesized **Fe-TMPP** (a) and that from the lyophilized sample (b; 10 mg in 10 mL of water, sonicated and kept for 24 h), showing no obvious change of the chemical composition.

			Size (d.nm):	% Intensity:	St Dev (d.nm):
Z-Average (d.nm):	611.8	Peak 1:	706.4	64.5	127.6
PdI:	0.585	Peak 2:	97.26	23.1	16.93
Intercept:	0.995	Peak 3:	19.48	12.4	2.816
Result quality :	Refer to quality report				

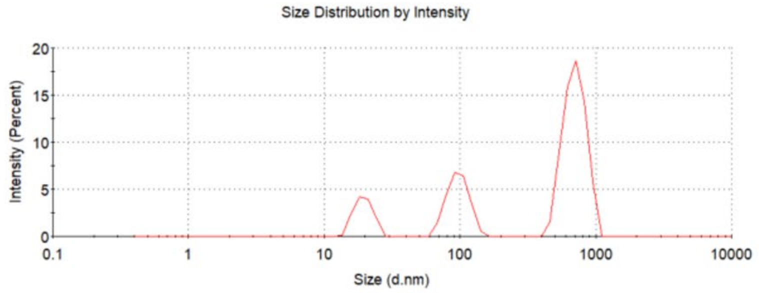

			Size (d.nm):	% Intensity:	St Dev (d.nm):
Z-Average (d.nm):	761.9	Peak 1:	381.2	71.0	51.37
PdI:	0.880	Peak 2:	39.05	22.2	6.500
Intercept:	1.03	Peak 3:	14.16	6.8	2.091
Result quality :	Refer to quality report				

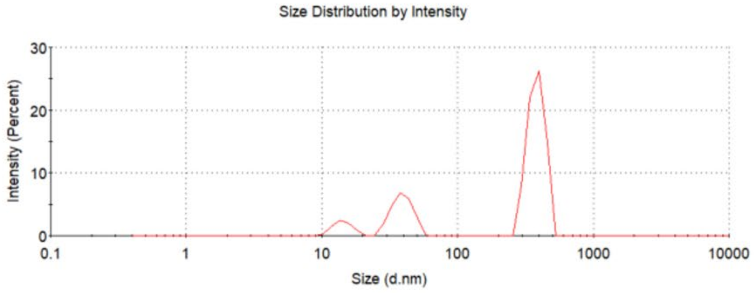

			Size (d.nm):	% Intensity:	St Dev (d.nm):
Z-Average (d.nm):	1459	Peak 1:	270.0	71.7	33.43
PdI:	0.899	Peak 2:	31.28	22.6	4.359
Intercept:	1.15	Peak 3:	12.43	5.7	1.548
Result quality :	Refer to quality report				

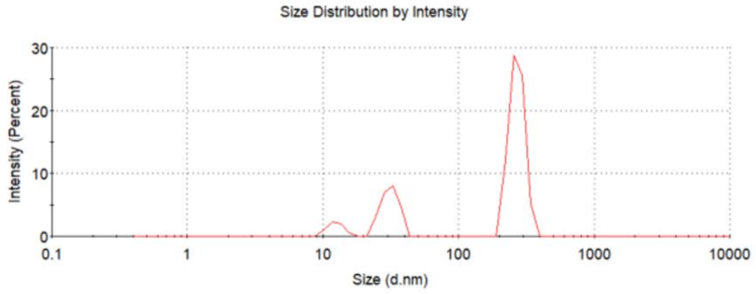

Figure S6. The DLS results of three **Fe-TMPP** aqueous solutions.

			Mean (mV)	Area (%)	St Dev (mV)
Zeta Potential (mV):	23.4	Peak 1:	23.4	100.0	5.96
Zeta Deviation (mV):	5.96	Peak 2:	0.00	0.0	0.00
Conductivity (mS/cm):	0.0214	Peak 3:	0.00	0.0	0.00

Result quality : Good

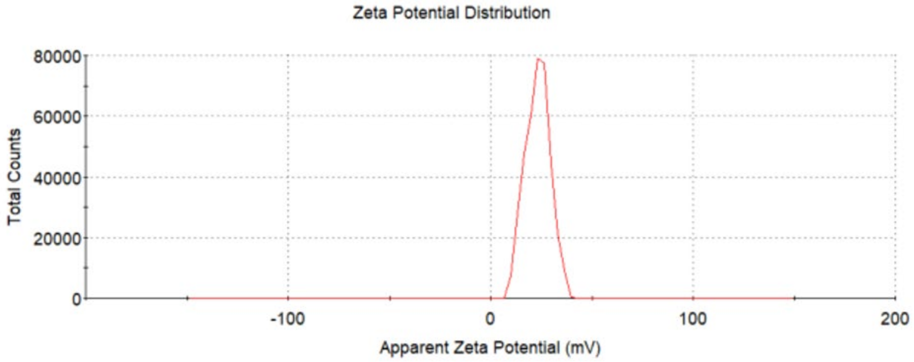

Figure S7. The zeta potential results of **Fe-TMPP** in aqueous solution.

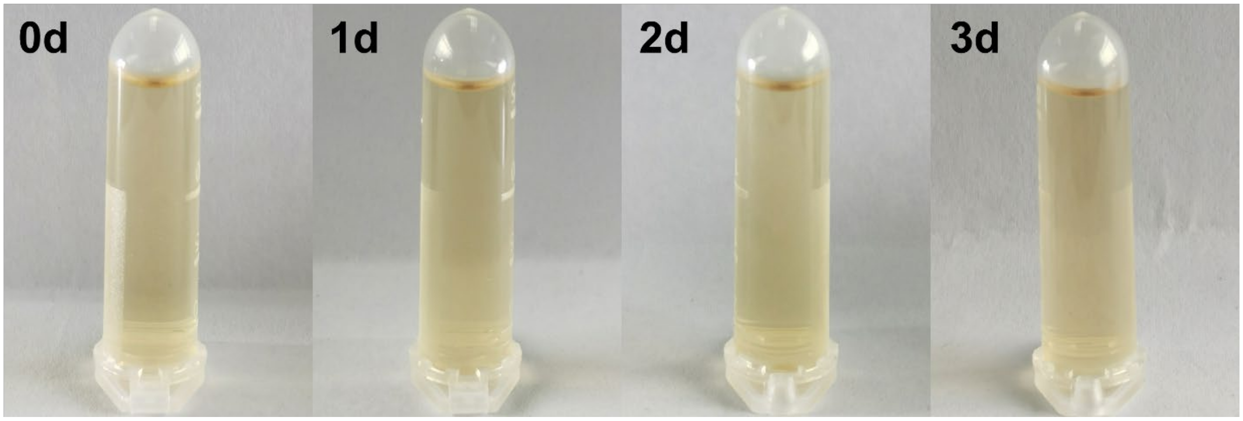

Figure S8. Pictures of **Fe-TMPP** dispersed in aqueous solution for 0–3 days.

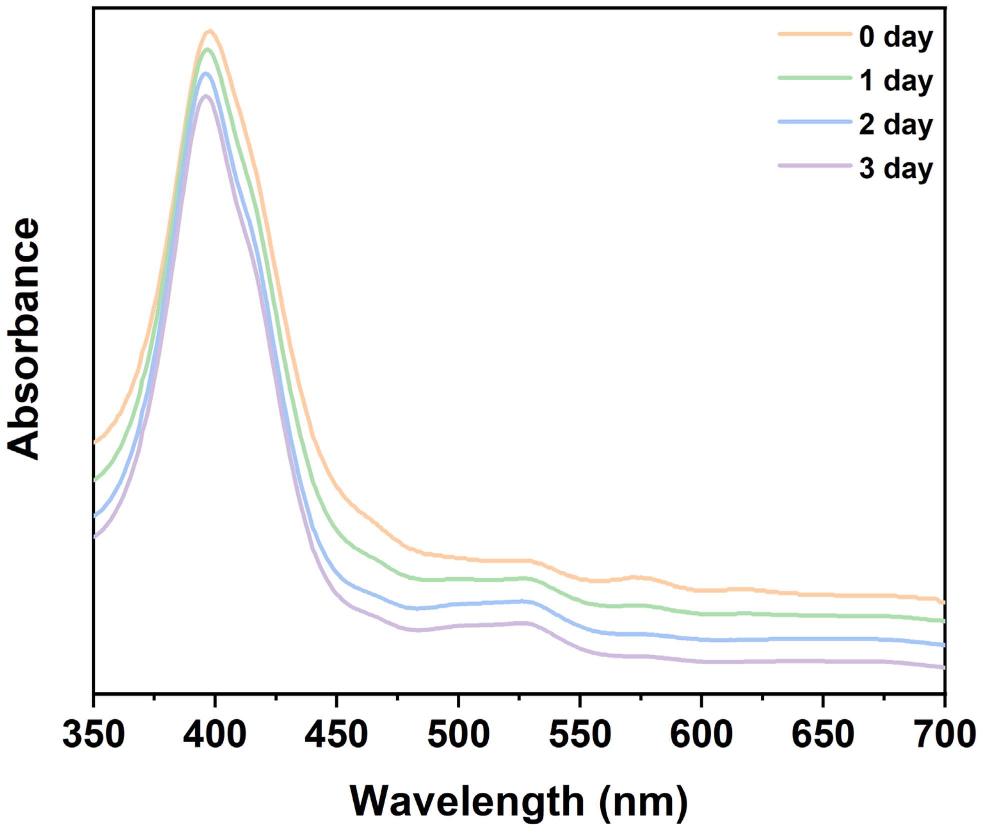

Figure S9. UV-Vis absorption spectra of **Fe-TMPP** dispersed in aqueous solution for 0–3 days.

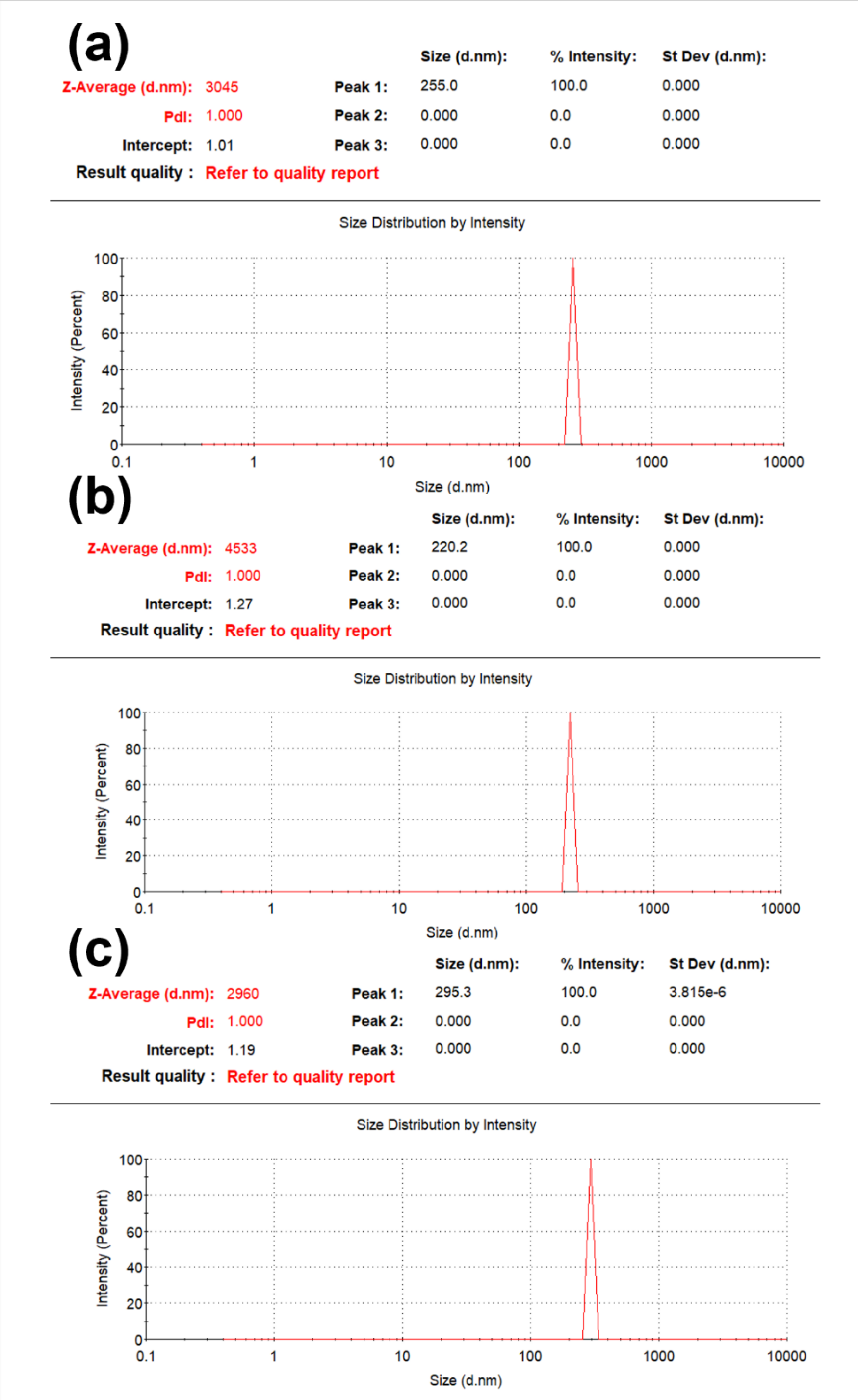

Figure S10. The DLS results of **Fe-TMPP** in PBS (0.1x) for 0 (a), 12 (b), and 24 hours (c).

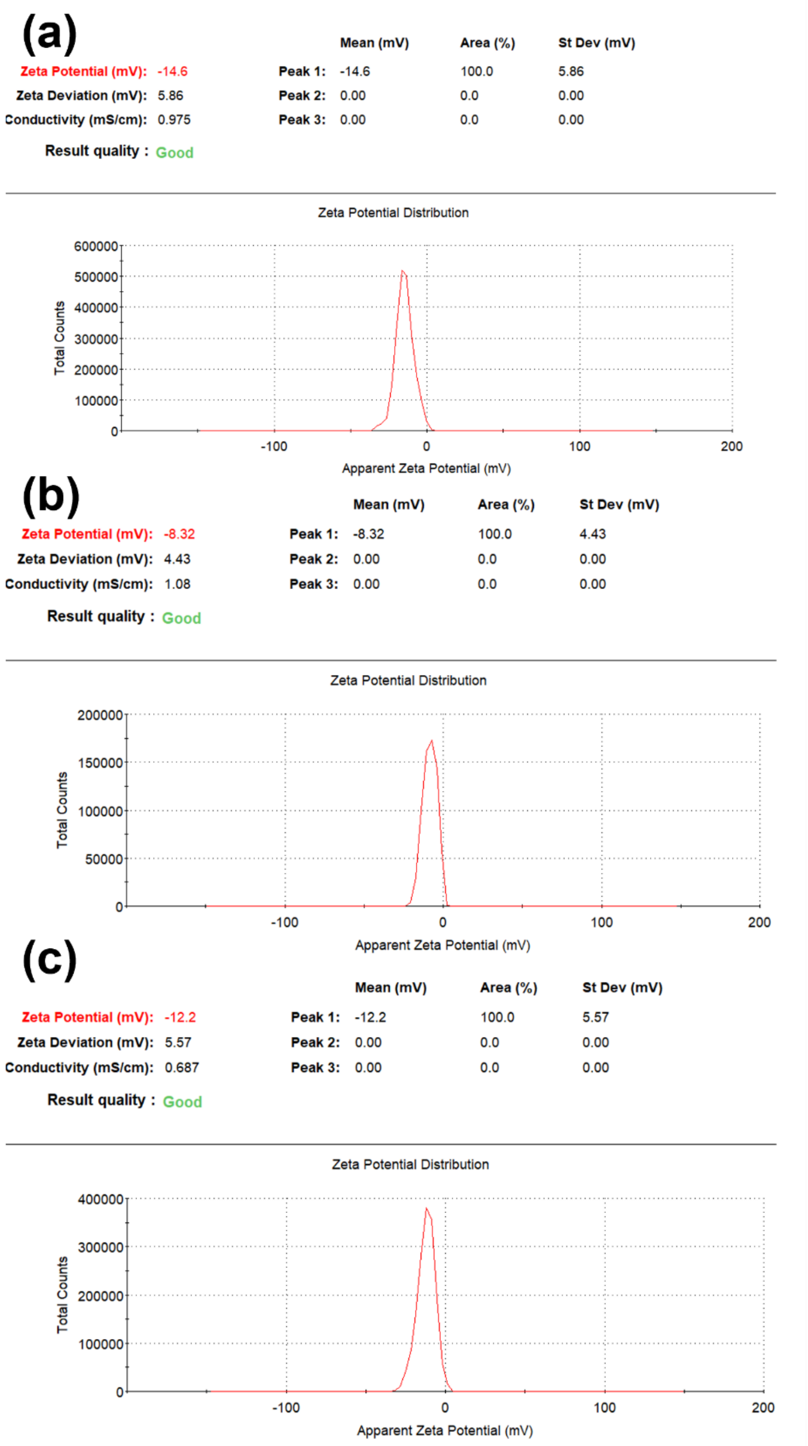

Figure S11. The zeta potential results of **Fe-TMPP** in PBS (0.1x) for 0 (a), 12 (b) and 24 hours (c).

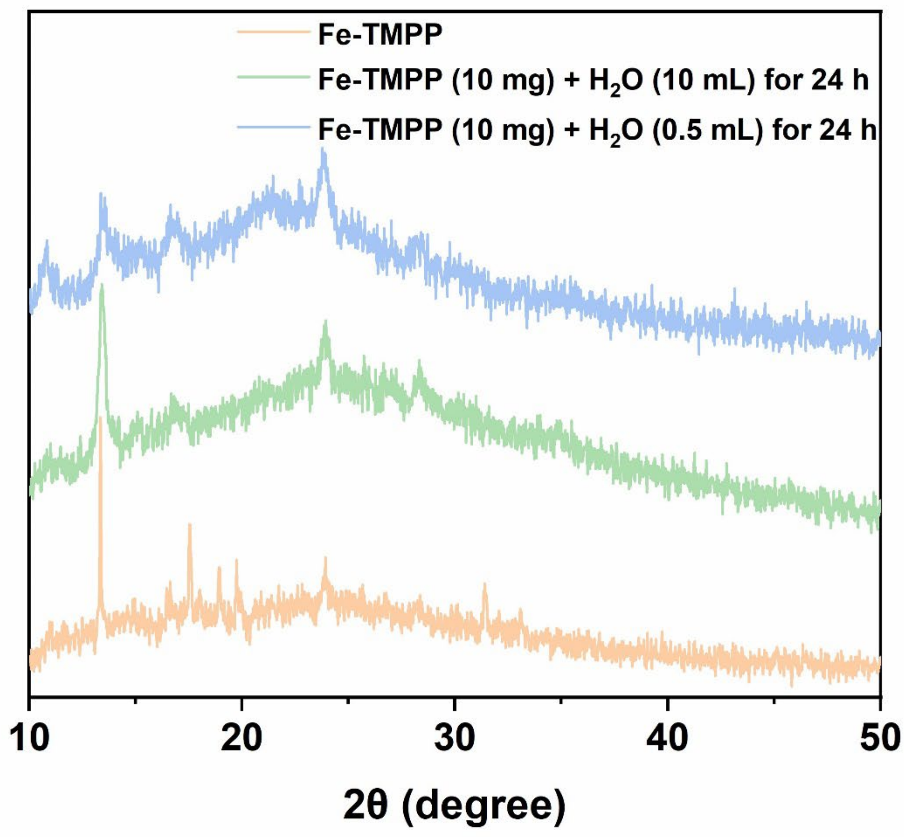

Figure S12. A comparison of the PXRD spectra of **Fe-TMPP** and its lyophilized samples after sonication and kept in different amounts of water for 24 h.

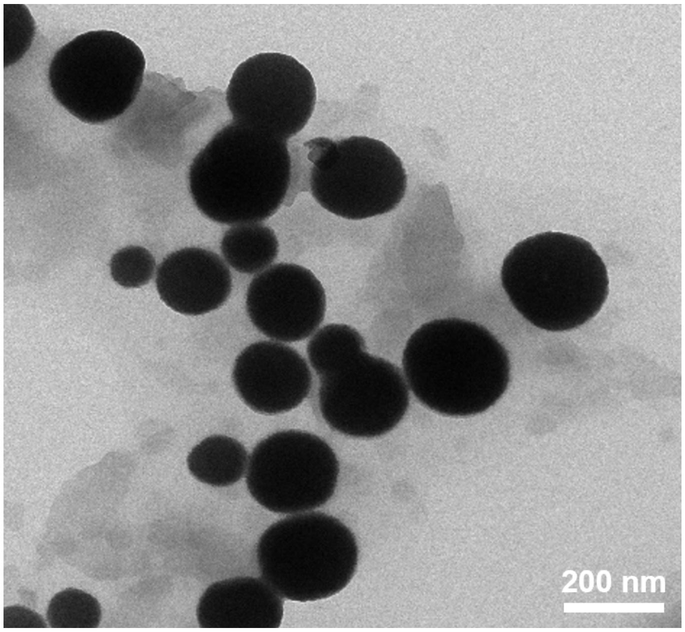

Figure S13. TEM image of **TMPP-F127**.

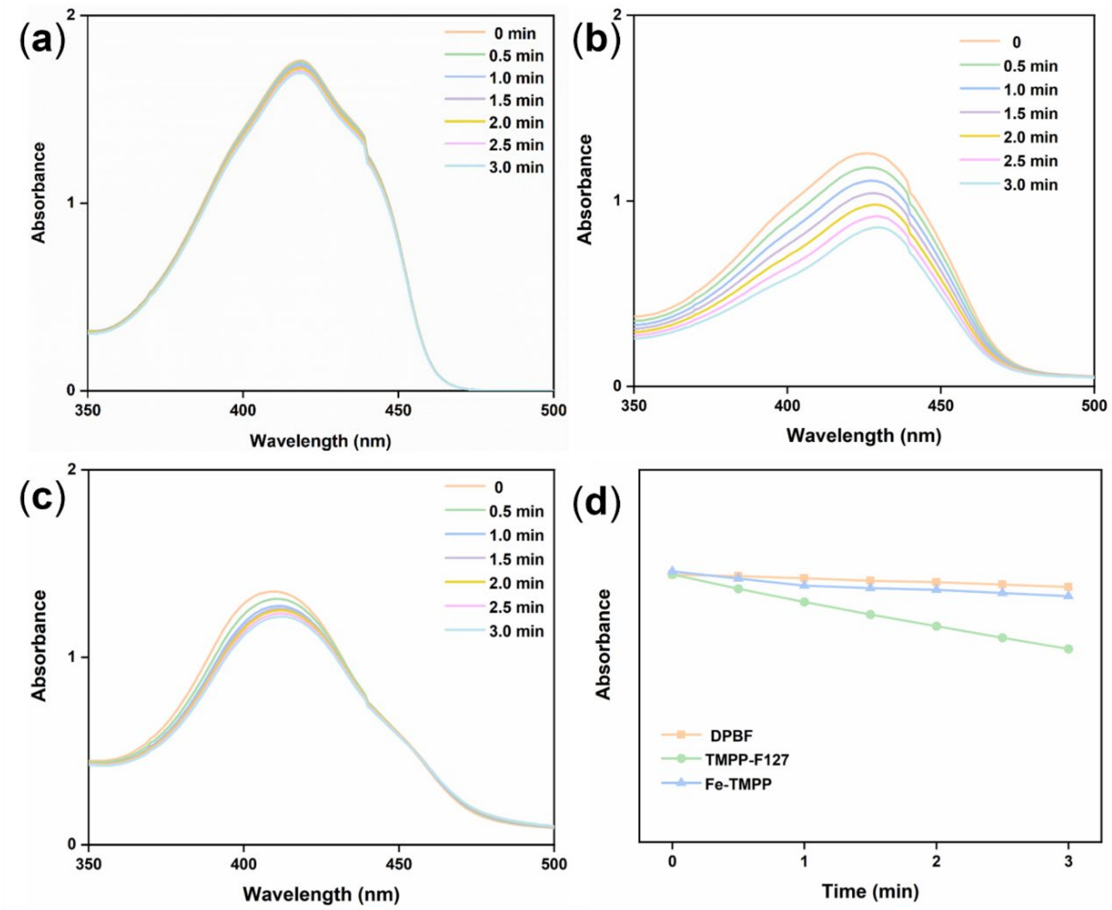

Figure S14. UV-Vis absorption spectra of DPBF (a) and its dispersion in **TMPP-F127** (b) and **Fe-TMPP** (c) aqueous solution with different illumination times. The changes of absorption intensity at 416 nm of DPBF as a function of irradiation time when incubated with **TMPP-F127** and **Fe-TMPP** using blank DPBF as a control (d).

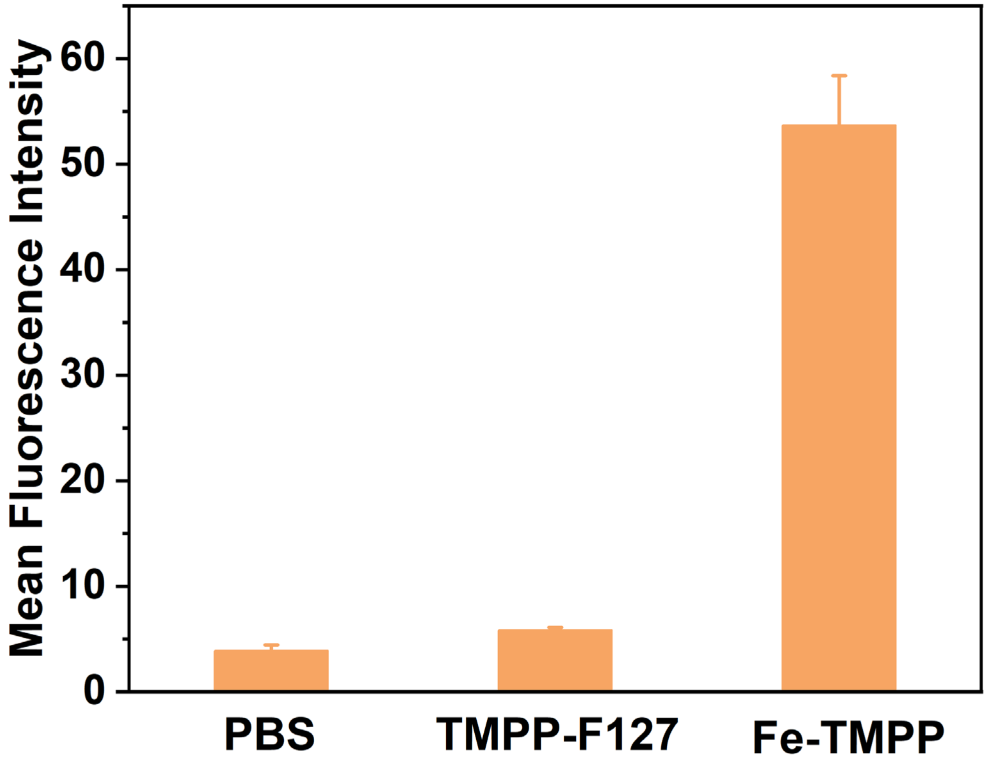

Figure S15. A comparison of fluorescence intensity of DCF when treated with **TMPP-F127**, **Fe-TMPP**, and PBS control.

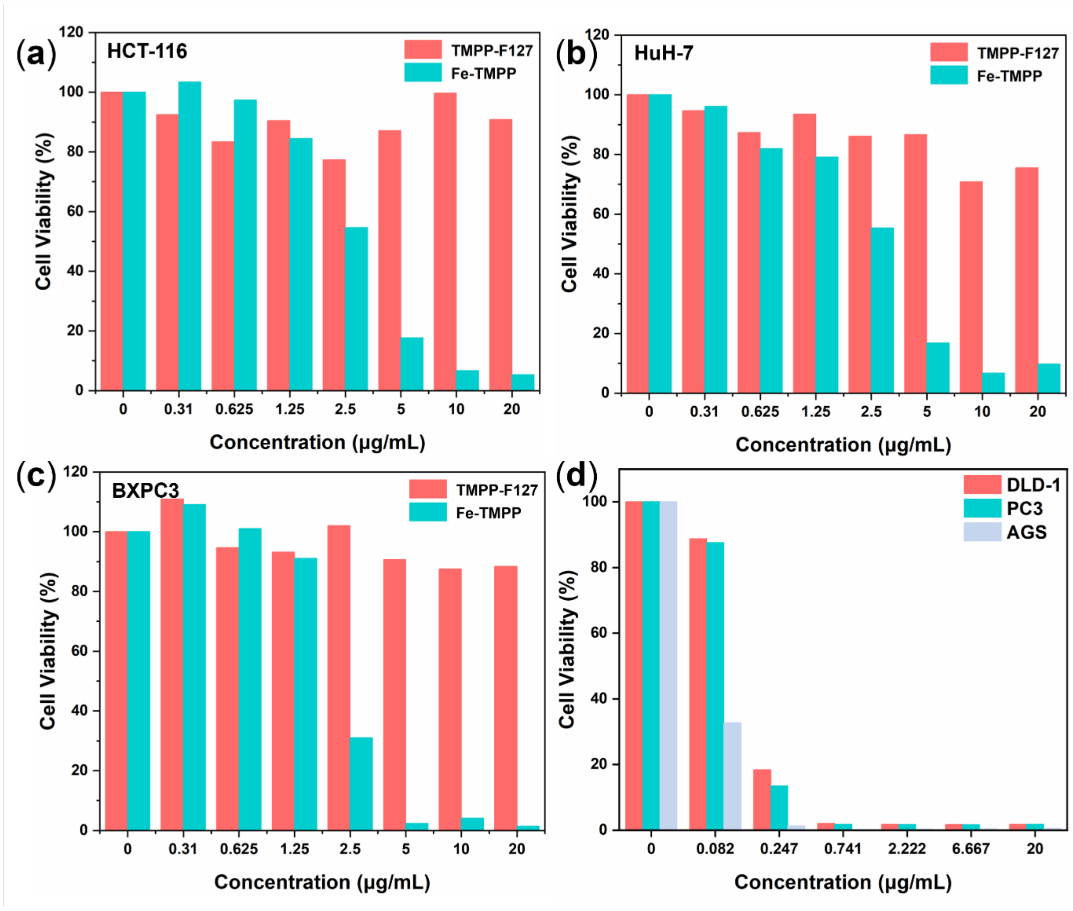

Figure S16. A comparison of the HCT-116 (a), HuH-7 (b), and BXPC3 (c) cell viabilities when treated with gradient concentrations of **TMPP-F127** and **Fe-TMPP**. The DLD-1, PC3, and AGS cells viabilities when treated with gradient concentrations of **Fe-TMPP** (d).

Table S1. Detailed cell development and drug incubation conditions.

Cell Line	Culturing Media	Culture Media Supplier	Cell Density	Incubation Time
4T1	RPMI 1640	Elabscience Crop.	5×10^3	24 h
HCT-116	DMEM	Procell Life Science & Technology Co., Ltd.	2×10^4	24 h
HuH-7	DMEM	Procell Life Science & Technology Co., Ltd.	2×10^4	24 h
BXPC3	RPMI 1640	Procell Life Science & Technology Co., Ltd.	2.5×10^3	72 h
DLD-1	RPMI 1640	Procell Life Science & Technology Co., Ltd.	2×10^3	72 h
PC3	RPMI 1640	Procell Life Science & Technology Co., Ltd.	2×10^3	72 h
AGS	Ham's F-12	Procell Life Science & Technology Co., Ltd.	2×10^3	72 h
